# *TREM2* Downregulation Disrupts Microglial Function and Synaptic Pruning Through RA/RARα Signaling: Mechanisms Underlying Autism‐Like Behaviors

**DOI:** 10.1002/pdi3.70024

**Published:** 2025-10-16

**Authors:** Min Lu, Kexin Bai, Yali Bai, Yuan Miao, Tingjiao Zhao, Yi Yang, Jie Chen, Ting Yang, Tingyu Li, Hua Wei

**Affiliations:** ^1^ Growth, Development and Mental Health Center of Children and Adolescents Children's Hospital of Chongqing Medical University Chongqing China; ^2^ Chongqing Key Laboratory of Child Neurodevelopment and Cognitive Disorders Chongqing China; ^3^ Ministry of Education Key Laboratory of Child Development and Disorders National Clinical Research Center for Child Health and Disorders Chongqing China

**Keywords:** autism spectrum disorder, microglia, myeloid cell trigger receptor 2, retinoic acid receptor α, synaptic pruning

## Abstract

Autism spectrum disorder (ASD) involves neuroimmune dysregulation and synaptic pruning defects. This study aimed to investigate the role of triggering receptor expressed on myeloid cells 2 (TREM2) in ASD pathogenesis and its link to retinoic acid (RA)/retinoic acid receptor α (RARα) signaling. Prefrontal cortex–specific knockdown of *TREM2* in rats induced core ASD‐like behaviors (e.g., social deficits), microglial hyperactivation, aberrant synaptic pruning, reduced serum soluble TREM2 (sTREM2) levels, and disrupted RA/RARα signaling. Oral RA supplementation (6 mg/[kg·day]) reversed these neuroimmune abnormalities and behavioral impairments. In vitro studies demonstrated that *TREM2* knockdown and RA supplementation induced RARα‐level alterations consistent with in vivo observations. These findings indicated that TREM2 deficiency was a key factor in the pathophysiology of ASD, mediated by the RA/RARα signaling pathway. Furthermore, serum sTREM2 might serve as a potential diagnostic biomarker for ASD. Collectively, these findings underscore the pivotal role of TREM2 in ASD pathogenesis and provide novel perspectives for diagnostic and therapeutic strategies.

## Graphical abstract

Downregulation of *TREM2* induces autism‐like behaviors in healthy rats by impairing microglial function and triggering aberrant synaptic pruning. RA/RARα signaling pathway modulates alterations in *TREM2* expression. Serum sTREM2 levels correspond to alterations in brain *TREM2* expression.

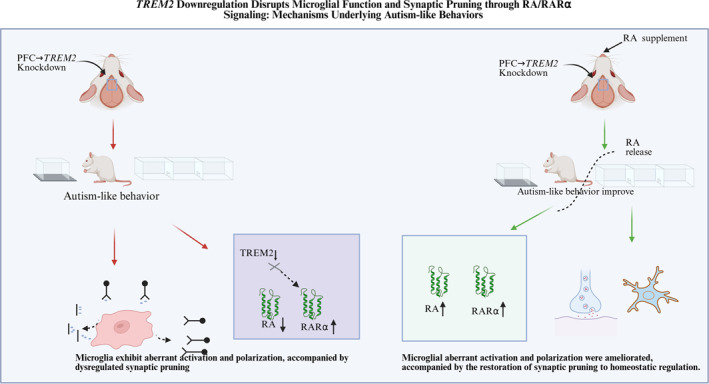

## 1 Introduction

Autism spectrum disorder (ASD) is a neurodevelopmental disorder characterized by social impairments, repetitive stereotyped behaviors, and restricted interests. It has become one of the fastest‐growing disorders worldwide [1]. Although contemporary research highlights the interplay of genetic and environmental factors in the pathogenesis of ASD, the etiology of ASD is complex and still incompletely understood [2]. Emerging evidence suggests an essential role of the neuroimmune system in ASD development, with immune‐regulating genes potentially influencing synaptic pruning and other neurodevelopmental processes [3].

Microglia are the resident immune cells of the central nervous system (CNS) involved in various essential functions, including neuronal development, myelin formation, and synaptic pruning and remodeling [4, 5]. Recent studies have reported activated microglia in the postmortem brains of patients with ASD [6]. Positron emission tomography/computed tomography scans have further revealed microglial activation in the frontal lobe and cerebellum of patients with ASD [7]. The transcriptomic analysis of postmortem brains revealed specific enrichment of microglia‐associated modules and immune response genes in the patients. The expression levels of these markers showed a significant inverse correlation with the dysregulation in neuronal splicing regulators, such as RNA‐binding Fox‐1 Homolog 1 (*RBFOX1*) isoforms. These findings indicated altered innate immune activation and disruptions in neuronal activity within the ASD pathogenesis [8]. Chronic neuroinflammation during early brain development activated microglia, leading to dendritic spine alterations and behaviors associated with ASD [9]. Collectively, these findings underscore the pivotal role of microglia in ASD pathogenesis. Understanding the mechanisms of microglial activation can provide valuable insights into ASD mechanisms and potential therapeutic strategies.

Triggering receptor expressed on myeloid cells 2 (TREM2), a member of the immunoglobulin superfamily, serves as a pattern recognition receptor within the innate immune system. It is primarily localized to microglia in the CNS, where its functional activities include modulation of neuroinflammatory responses [10]. TREM2 is crucial for regulating microglial activity and function, neuronal protection, repair, and microglia‐dependent synaptic clearance [11]. The extracellular domain of TREM2 is cleaved by *α*‐secretase, releasing soluble TREM2 (sTREM2) [12], which can be detected in cerebrospinal fluid (CSF) and blood. A limited number of studies have explored the relationship between TREM2 and ASD. Population studies have revealed significantly reduced TREM2 protein levels in the postmortem brain tissues of patients with autism, with a negative correlation between TREM2 levels and autism severity scores [13]. This suggests that aberrant *TREM2* expression may contribute to ASD pathogenesis.

Our previous animal model studies [14] revealed reduced *T* *REM2* expression in the prefrontal cortex (PFC) of rats exhibiting autism‐like behaviors. This reduction impaired microglial synaptic pruning in these rats. These findings suggested the involvement of the TREM2 signaling pathway in the etiology of ASD. Concurrently, we discovered that retinoic acid receptor α (RARα) can bound to the promoter region of the *TREM2* gene [15], regulating its transcription and ameliorating autism‐like behaviors in valproic acid (VPA) model rats. Vitamin A, is a micronutrient essential for physiological functions. Animal studies have demonstrated that vitamin A deficiency induces synaptic structural abnormalities in rats [16]. However, in human physiology, vitamin A does not directly exert its biological effects in its native form. Retinoic acid (RA), which is the active metabolite of vitamin A, mediates its biological functions through binding to RARs, which subsequently regulate transcriptional cascades crucial for CNS development [17]. Studies have reported reduced RA levels in pediatric patients with ASD, with an inverse correlation between RA levels and ASD symptom severity [18]. RARα is highly expressed in the brain, particularly in the PFC and hippocampus [19]. Studies suggest that the RA/RARα signaling pathway may regulate microglial activation [20]; however, the precise mechanisms remain unclear.

We performed *T* *REM2* knockdown (KD) in wild‐type (WT) rats to further validate the previously identified regulatory relationship between *RARα* and *TREM2*. Initial assessments focused on microglial cell activation profiles and polarization states, synaptic alterations, and behavioral deficits within the PFC. Subsequently, RA was supplemented following *TREM2* KD, and the aforementioned assessments were repeated. Complementary in vitro experiments using cultured BV2 microglial cells were conducted to assess the changes in *RARα* expression after *TREM2* KD and subsequent RA treatment. The results demonstrated that *TREM2* reduction elicited the expected pathological outcomes, including aberrant microglial activation and polarization, impaired synaptic pruning, and manifestation of autism‐like behaviors. The gene *TREM2* KD also resulted in concomitant upregulation of *RARα* expression. RA supplementation effectively rescued these *TREM2* deficiency‐induced abnormalities by normalizing microglial activity and synaptic pruning deficits and significantly ameliorating autism‐like behavioral phenotypes. In vitro experiments using BV2 microglial cells demonstrated that *TREM2* knockdown with subsequent RA supplementation recapitulated the changes in *RARα* expression changes observed in vivo, supporting the role of RA/RARα signaling in modulating *TREM2* downregulation. These findings collectively suggest that downregulation of *TREM2* may alter microglial activation and synaptic pruning through the RA/RARα signaling pathway, ultimately leading to autism‐like behaviors. This finding provides a foundation for exploring novel therapeutic targets and intervention strategies for ASD.

## 2 Materials and Methods

### 2.1 Animals

Sprague Dawley (SD) rats were procured from the Animal Center of Chongqing Medical University and maintained in the specific‐pathogen‐free‐grade animal breeding room of the Animal Center of Children's Hospital of Chongqing Medical University in Chongqing, China. They were housed under a 12‐h light/12‐h dark cycle. The experimental protocol was approved by the animal ethics committee of the Children's Hospital of Chongqing Medical University (CHCMU‐IACUC20240111004).

### 2.2 Experimental Model and Grouping

WT SD female rats, procured at the age of 8 weeks were mated with male rats. The date of birth of the offspring was recorded. The following of two portions of the experiment were conducted at postnatal day 14 (PND 14), after injecting empty adeno‐associated virus and *TREM2*‐kd adeno‐associated virus into the lateral ventricle.

**FIGURE 1:**
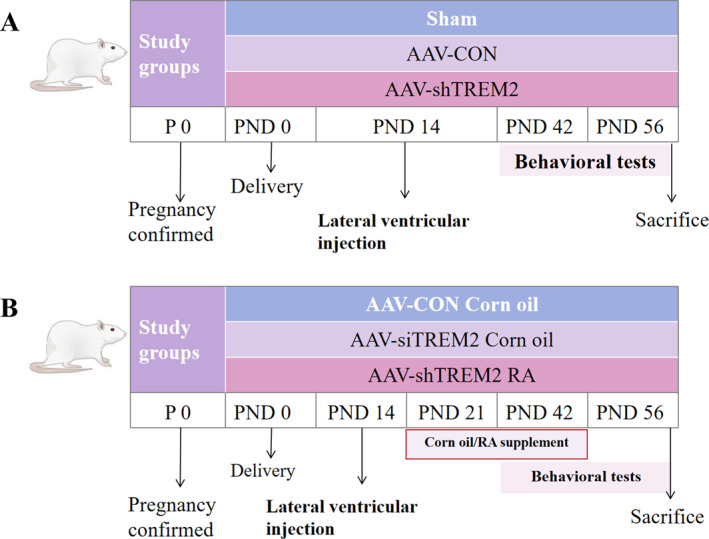
A schematic diagram of the experimental protocol. (A) Experimental diagram of Sham, AAV‐CON and AAV‐shTREM2 groups. Sham: Needle track insertion only (no injection).​ AAV‐CON: Injection of control AAV.​ AAV‐shTREM2: Injection of AAV encoding shRNA for *TREM2* kd. Injection parameters for AAV‐CON and AAV‐shTREM2 groups: volume 5 μL; rate 0.5 μL/min; post‐injection needle retention 5 min. (B) Schematic diagram of experiments of AAV‐CON Corn oil, AAV‐shTREM2 Corn oil, and AAV‐shTREM2 RA groups. In AAV‐CON Corn oil group, corn oil (2.4 mL/kg·day) was administered once a day for 3 weeks; in AAV‐shTREM2 Corn oil group, at the same dose and frequency; in AAV‐shTREM2 RA group, retinoic acid (6 mg/kg·day) was administered at the same dose and frequency. Each group of 16 individuals, with PND 0 on the day of birth, were assessed for behavioral testing at PND 42 and brain tissue was collected at PND 56 for further experiments. AAV, Adeno‐associated virus; AAV‐CON, control empty virus; AAV‐shTREM2, AAV particles containing TREM2; kd, knockdown; PND, postnatal days; Sham, no injection.

Experiment 1: PND 14 littermates were assigned to three groups (a sham‐operated group, an empty carrier virus‐injection group, and a *TREM2*‐kd adeno‐associated virus injection group). The animals were weaned on PND 21 and behavioral experiments were completed on PND 42 (Figure 1A).

Experiment 2: PND 14 littermates received intracerebroventricular (ICV) injections of either control adeno‐associated virus (AAV) (AAV‐CON) or *TREM2*‐kd AAV (AAV‐shTREM2). On PND 21, the littermates were supplemented with corn oil or RA (6 mg/kg, Sigma, Darmstadt, Germany) via oral gavage. The littermates were divided into three groups:

1. AAV‐CON Corn oil: ICV control AAV followed by corn oil gavage.
2. AAV‐shTREM2 Corn oil: ICV *TREM2*‐kd AAV followed by corn oil gavage.
3. AAV‐shTREM2 RA: ICV *TREM2*‐kd AAV followed by RA gavage.

RA/Corn oil was administered daily for 3 weeks. The dosage and route of administration were based on the previous findings [21]. The behavioral testing was conducted on PND 42 (Figure 1B). Male rats were used in this experiment to eliminate any potential sex‐based differences.

### 2.3 AAV Preparation and Brain Stereotactic Injection

The *TREM2*‐kd AAV and null‐loaded virus required for this experiment were provided by Sangon Biotech Co. Ltd. (Shanghai, China). The titer of AAV‐shTREM2 virus and AAV‐CON was 6.83 × 10^12^ and 2.02 × 10^13^ vg/mL, respectively. The virus was injected into the left lateral ventricle. The virus was injected into the left lateral ventricle at a rate of 0.5 μL/min with an automated stereotactic injector (RWD, Shenzhen, Guangdong, China). The injection was performed at a depth of 1.5 mm near the midline, 0.8 mm posterior to the cerebrospinal membrane, and 2.5 mm below the skull for 10 min (total volume: 5 μL) [22].

### 2.4 Behavioral Tests

#### 2.4.1 Open‐Field Test and Self‐Grooming Experiments

The open‐field experiments were conducted in a black box with a volume (48 × 48 × 60 cm^3^). The floor of the box was divided into two zones: the central zone and the peripheral zone. The rats were placed in the central zone at the beginning of the experiment and allowed to move freely for 5 min. The time spent in each zone and the total distance traveled were recorded using the ANY‐Maze video tracking system (ANY‐Maze6.3, Chicago, Illinois, USA). The self‐combing test quantified the time the rat spent in combing its fur with its mouth or paws, which could be used to indicate repetitive stereotyped behaviors. The self‐combining time was recorded by a trained professional, and the statistician was blinded to the grouping information.

#### 2.4.2 Three‐Chamber Test

The three‐chamber social test was performed using a 60 × 40 × 30 cm^3^ apparatus to sequentially assess rodent social behavior across three 5‐min phases: (1) habituation: the rats freely explored the empty chambers; (2) sociability: the rats interacted with a novel conspecific (stranger 1) versus an inanimate object in opposing chambers, with social preference quantified using the ANY‐Maze tracking system; and (3) social novelty preference: the rats explored stranger 1 (now familiar) versus a novel conspecific (stranger 2), with differential investigation time indexing social recognition memory. This protocol objectively measured baseline sociability (social > object interaction) and cognitive flexibility (novel > familiar social exploration) based on automated chamber occupancy and direct sniffing time (snout ≤ 3–5 cm from the target).

### 2.5 Tissue Collection and Experiments

After the completion of behavioral studies, the brains of rats aged 6–8 weeks were harvested. The rats were anaesthetized, and the PFC tissues were excised by severing the head and stored in a −80°C for subsequent experiments.

#### 2.5.1 Western Blotting

Total brain tissue proteins were extracted using Radio Immuno Precipitation Assay (RIPA) buffer lysate (KeyGEN BioTECH, Nanjing, Jiangsu, China). Western blotting experiments were performed following the methodology described previously [21]. The protein loading amount was 30 μg per sample. The antibodies used in the experiments are detailed in Supporting Information S1: Table S1.

#### 2.5.2 Real‐Time Quantitative Polymerase Chain Reaction (RT‐qPCR)

RNA was extracted from the PFC using an RNA extraction kit (BioFlux, Beijing, China), followed by reverse transcription to cDNA. Subsequently, RT‐qPCR experiments were conducted using SYBR Green Mix (TaKaRa, Shijodori, Shimogyo‐ku, Japan) and a CFX96 detection system. The list of primers used in the study is provided in Supporting Information S1: Table S2.

#### 2.5.3 Enzyme‐Linked Immunosorbent Assay (ELISA)

The alterations in the levels of RA (NEWA, Shanghai, China), IL‐1β (ABclonal, Wuhan, Hubei, China), TNF‐α (NeoBioscience, Shenzhen, Guangdong, China), IL‐4 (ABclonal, Wuhan, Hubei, China), and IL‐10 (ABclonal, Wuhan, Hubei, China) in the PFC were quantified using ELISA kits. The changes in the serum levels of sTREM2 (Finest, Wuhan, Hubei, China) and inflammatory factors were also assessed. The experiments were conducted following the instructions of the reagent vendors.

#### 2.5.4 Cell Culture

BV2 mouse microglial cells were cultured in high‐glucose Dulbecco's modified Eagle's medium (Gibco, Waltham, Massachusetts, USA) supplemented with 10% fetal bovine serum (Gibco, Waltham, Massachusetts, USA). The cells were maintained in a humidified incubator at 37°C in the presence of 5% CO_2_. They were transfected with siRNA using Lipofectamine 2000 (Invitrogen) following the manufacturer's protocol. The cultures were treated with 4 μM RA 24 h after transfection. Immunofluorescence (IF) staining was performed 24 h following RA administration.

#### 2.5.5 IF Staining

Rats underwent transcardial perfusion under deep anesthesia. Brains were immersion‐fixed in 4% paraformaldehyde (PFA), cryoprotected in sucrose, embedded in optimal cutting temperature (OCT) compound, and coronally sectioned at 30 μm using a cryostat (RWD, Shenzhen, Guangdong, China). BV2 cells were fixed in 4% PFA (30 min, RT). Both brain sections and BV2 cells were permeabilized with PBS/0.3% Triton X‐100, blocked with PBS/5% BSA for 1 h and then immunolabeled with species‐specific primary antibodies at 4°C overnight and fluorophore‐conjugated secondary antibodies. The nuclei were counterstained with DAPI in antifade mounting media. Confocal imaging (Nikon, Tokyo, Japan) was performed with 10X and 20X objectives. Microglial quantification and morphological analysis were conducted using Image J software (NIH, Bethesda, Maryland, USA) [23].

#### 2.5.6 Sholl Analysis and Golgi Staining

Following image analysis with Image J software, concentric circles were drawn around the cytosol, at 1 μm intervals. The number of intersections between the concentric circles and the dendrites at 1‐μm intervals was then estimated. The experiments were conducted using a Golgi staining kit FD Rapid Golgi Stain Kit (Fdneurotech, Columbia, Maryland, USA), following the instructions outlined in the laboratory manual [21]. The microscopic analysis employed a 40X objective. Considering the inherent resolution constraints of Golgi staining under light microscopy for detailed dendritic spine morphology, this study focused exclusively on quantifying spine density. The morphometric analysis of spine parameters (e.g., length, width, and subtype classification) was not performed.

#### 2.5.7 Statistical Analysis

The data were analyzed using GraphPad Prism software. Continuous variables with a normal distribution were presented as the mean ± standard error of the mean. Multiple comparisons were performed using one‐way and two‐way analysis of variance followed by the Tukey *post‐hoc* test. A *p* value less than 0.05 indicated a statistically significant difference.

## 3 Results

### 3.1 Successful *TREM2* Kd Was Achieved, and Rats Subjected to *TREM2* Kd Exhibited Autism‐Like Behavioral Phenotypes

**FIGURE 2:**
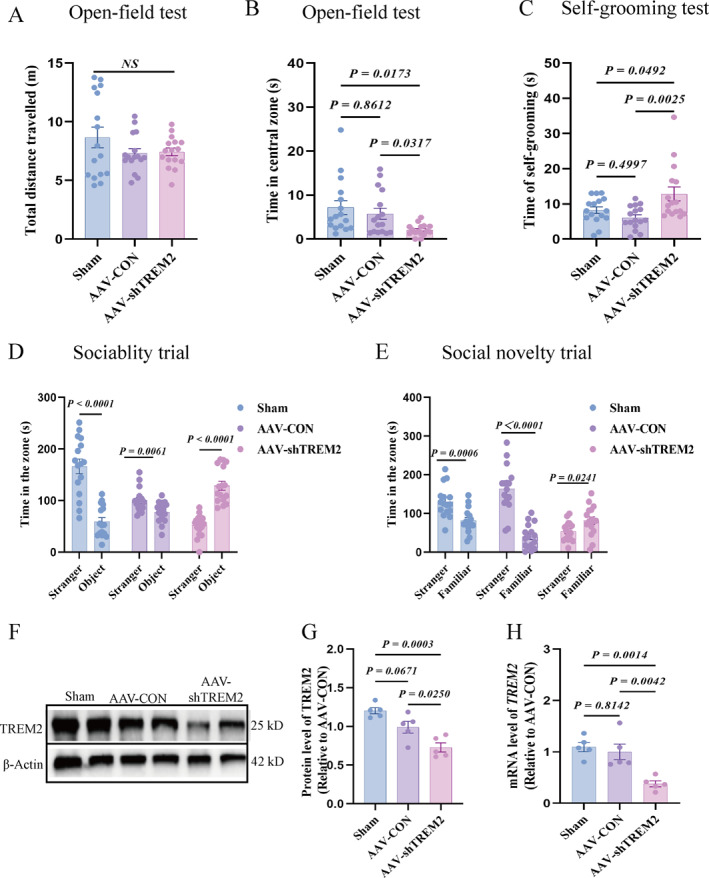
Autistic‐like behaviors and down‐regulated *TREM2* expression in the PFC of AAV shRNA knock‐down *TREM2* rats. (A) The total distance traveled in the open‐field test (*n* = 16/group 95% CI “−0.6826 to 3.375”, “−0.7991 to 3.258”, “−2.145 to 1.912”). (B) The time spent in the central zone in the open‐field test (*n* = 16/group 95% CI “−3.692 to 6.529”, “0.8428 to 9.420”, “0.2889 to 7.136”). (C) The time spent for self‐grooming in the open‐field test (*n* = 16/group 95% CI “−2.447 to 6.736”, “−9.196 to −0.01309”, “−11.34 to −2.157”). (D) The social interaction in the three‐chamber test (a stranger rat *vs.* an object) (*n* = 16/group 95% CI “78.30 to 135.9”, “−6.146 to 51.42”, “−106.1 to −48.53”). (E) The recognition of social novelty in the three‐chamber test (a stranger rat *vs.* a familiar rat) (*n* = 16/group 95% CI “14.79 to 82.78”, “89.14 to 157.1”, “−61.42 to 6.571”). (F–G) Representative western blot and quantification analysis of TREM2 protein in the PFC from the Sham, CON, and shTREM2 groups, as normalized to *β*‐Actin (*n* = 5/group 95% CI “−0.3634 to 0.2791”, “0.07919 to 0.7217”, “0.1214 to 0.7639”); (H) The mRNA level of *TREM2* in the PFC from the Sham, CON, and shTREM2 groups, as detected using RT‐QPCR and normalized to *GAPDH* (*n* = 5/group 95% CI “−0.3139 to 0.5025”, “0.3071 to 1.124”, “0.2128 to 1.029”). The values were displayed as the mean ± SEM (A–E, G–H). Ordinary one‐way ananlysis of ANOVA was used (A–E, G–H). AAV, adeno‐associated virus; AAV‐CON, control empty virus; AAV‐shTREM2, AAV particles containing *TREM2*; NS, not significant; PFC, prefrontal cortex; Sham, no injection.

Various behavioral tests were conducted to assess the behavioral changes after *TREM2* kd. The results revealed no statistically significant difference in the total distance traveled in the AAV‐shTREM2 group compared with the control group (Figure 2A), indicating that reduced TREM2 levels did not impair general locomotor abilities. However, the AAV‐shTREM2 group exhibited a significant reduction in time spent in the central area (Figure 2B) and an increase in self‐grooming time (Figure 2C). These findings suggested diminished exploratory behavior in novel environments and heightened engagement in repetitive stereotyped behaviors. Furthermore, the rats in the AAV‐shTREM2 group spent more time in the toy and rat familiarization areas during the socialization and social novelty tests (Figure 2D,E), indicating social deficits and reduced interest in novelty. Overall, these behavioral observations indicated that *TREM2* kd induced core autism‐like symptoms, including impaired social interactions and increased stereotypic behaviors. Furthermore, successful *TREM2* kd was confirmed through protein and mRNA expression analysis in the PFC of rats in the experimental groups (Figure 2F–H).

### 3.2 Kd of *TREM2* Triggered Aberrant Microglial Activation and Dysregulated Polarization

**FIGURE 3:**
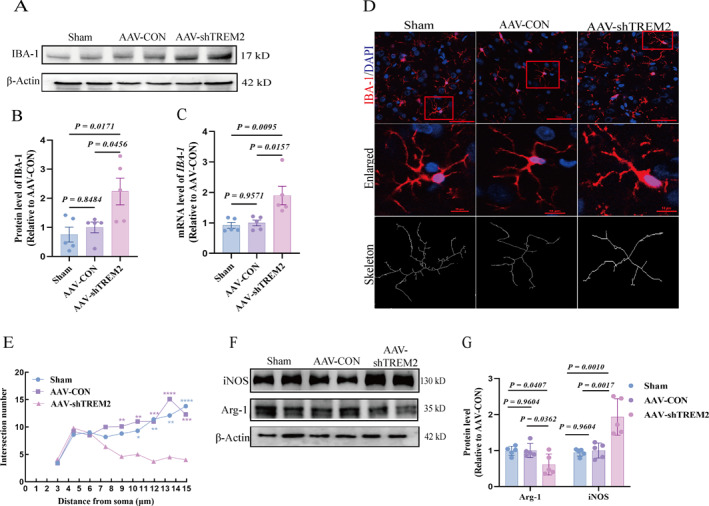
The kd of *TREM2* elicited aberrant microglial activation and polarization in the PFC of rats. (A–C) IBA‐1 protein and mRNA expression levels of *IBA‐1* in PFC of three groups (*n* = 5/group 95% CI of protein: “−1.461 to 0.9612”, “−2.695 to −0.2732”, “−2.445 to −0.02353”; 95% CI of RNA: “−0.8028 to 0.6490”, “−1.705 to −0.2534”, “−1.628 to −0.1765”). (D) Representative immunofluorescence staining and skeleton images of IBA‐1 in PFC of three groups. Scale bar = 50 μm, scale bar (Enlarged) = 10 μm. (E) Degree of microglia complexity. (F–G) Representative plots and statistical analysis plots of the iNOS and Arg‐1 protein blots in the PFC, internal reference is *β*‐Actin (*n* = 5/group 95% CI of iNOS: “−0.5976 to 0.4873”, “−1.531 to −0.4463”, “−1.476 to −0.3912”; 95% CI of ARG‐1: “−0.3614 to 0.3438”, “0.01536 to 0.7206”, “0.02413 to 0.7293”). All values are shown as the mean ± SEM (B–C, G); one‐way analysis of ANOVA was used (B–C, G). AAV, adeno‐associated virus; AAV‐CON, control empty virus; AAV‐shTREM2, AAV particles containing *TREM2*; kd, knockdown; PFC, prefrontal cortex; Sham, no injection. *p < 0.05; **p < 0.01; ***p < 0.001; ****p < 0.0001.

We analyzed the expression of the microglial marker ionized calcium‐binding adaptor molecule 1 (IBA‐1) to investigate the impact of *TREM2* kd on microglial activity. Both IBA‐1 protein and mRNA levels of *IBA‐1* were significantly increased following *TREM2* kd (Figure 3A–C). Immunofluorescence revealed increased IBA‐1^+^ microglial density and enhanced fluorescence intensity (Figure S1A,B,C). Microglia displayed hypertrophic somata, shortened processes (Figure 3D), and reduced arborization complexity via Sholl analysis (Figure 3E), including decreased branch endpoints, total branches, and branch length (Figure S3A–D). The phenotypic profiling demonstrated microglial polarization toward a pro‐inflammatory state: inducible nitric oxide synthase (iNOS) and *CD68/CD86* were upregulated, whereas arginase‐1 (Arg‐1) and *CD206* were downregulated (Figure 3F,G and Figure S3E–G). Consistent with this shift, elevated IL‐1β/TNF‐α and reduced IL‐4/IL‐10 were observed in the PFC (Figure S3H–K). Collectively, *TREM2* deficiency drove microglial hyperactivation and inflammatory dysregulation.

### 3.3 *TREM2* Kd Induced Synaptic Protein Dysregulation in WT Rats Through the Complement System, Independent of Alterations in Neuronal Population

**FIGURE 4:**
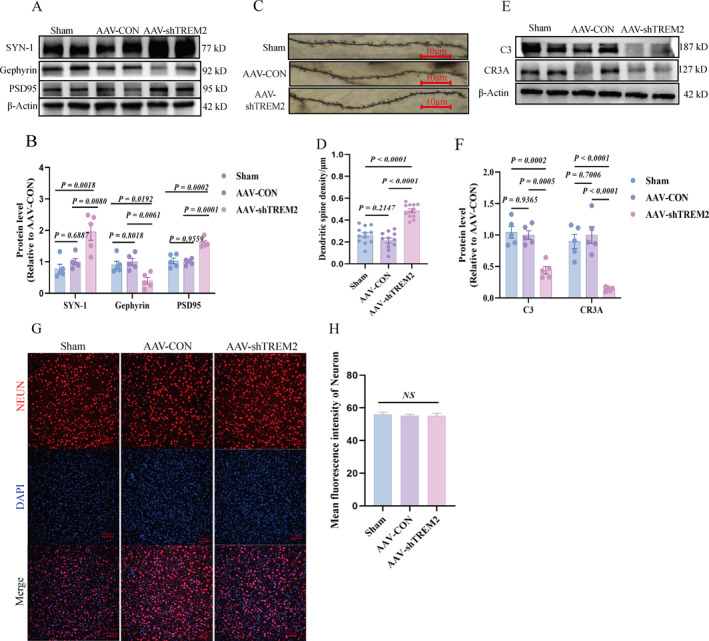
*TREM2* downregulation induces aberrant synaptic pruning and dysregulated synaptic protein expression via the complement system instead of neuronal number change. (A–B) Representative protein blotting and quantitative analysis of synaptic proteins in PFC of three groups of rats, the internal reference is *β*‐Actin (*n* = 5/group 95% CI of SYN‐1: “−0.1667 to 0.08716”, “−0.3426 to −0.08868”, “−0.3028 to −0.04890”; 95% CI of Gephyrin: “−1.218 to 0.7474”, “0.1979 to 2.164”, “0.4334 to 2.399”; 95% CI of PSD95: “−0.3312 to 0.4109”, “−1.207 to −0.4648”, “−1.247 to −0.5047”). (C) Representative images of dendritic spines in PFC brain of three groups of rats (*n* = 12 slices from 4 rats per group), scale bar = 10 μm. (D) Dendritic spine analysis diagram. (E–F) Representative diagram and statistical analysis diagram of protein imprinting of complement C3/CR3A in brain PFC, the internal reference is *β*‐Actin (*n* = 5/group 95% CI of C3: “−0.3442 to 0.4703”, “0.4705 to 1.285”, “0.4075 to 1.222”; 95% CI of CR3A: “−0.4831 to 0.2800”, “0.3765 to 1.140”, “0.4780 to 1.241”). (G–H) Representative immunofluorescence micrographs and quantitative analysis of neuronal density in the PFC across three experimental rat cohorts (*n* = 12 slices from 4 rats per group), scale bar = 100 μm. All values are expressed as mean ± SEM (B, D, F, H); two‐way analysis of variance (B) and one‐way analysis of variance were used (D, F, H). AAV, adeno‐associated virus; AAV‐CON, control empty virus; AAV‐shTREM2, AAV particles containing *TREM2*; PFC, prefrontal cortex; Sham, no injection.

Microglia are key mediators of synaptic pruning. We analyzed the expression of key synaptic markers to explore the impact of *TREM2* kd on synaptic proteins and dendritic spine density. The *TREM2* kd significantly increased dendritic spine density (Figure 4C,D) and altered synaptic protein expression, with presynaptic synapsin‐1 (SYN‐1) and postsynaptic excitatory PSD95 being upregulated while inhibitory gephyrin was downregulated (Figure 4A,B). These changes coincided with reduced complement components C3 and CR3A in the PFC (Figure 4E,F), indicating impaired complement‐mediated synaptic pruning. The colocalization analysis confirmed diminished interactions between IBA‐1^+^ microglia and PSD95, as well as reduced C1q‐PSD95 coupling (Figure S2C–F), further supporting disrupted microglial synaptic clearance. The neuronal density remained unchanged across groups (Figure 4G,H), confirming that synaptic alterations were driven by TREM2‐dependent pruning defects rather than neuronal loss. These findings suggested that reduced TREM2 levels led to abnormal synaptic protein expression and potentially impaired synaptic pruning through disrupting the complement system.

### 3.4 RA Levels Decreased, *RARα* Expression Increased, and Serum sTREM2 Level Was Reduced in the PFC of sh*TREM2* Rats

**FIGURE 5:**
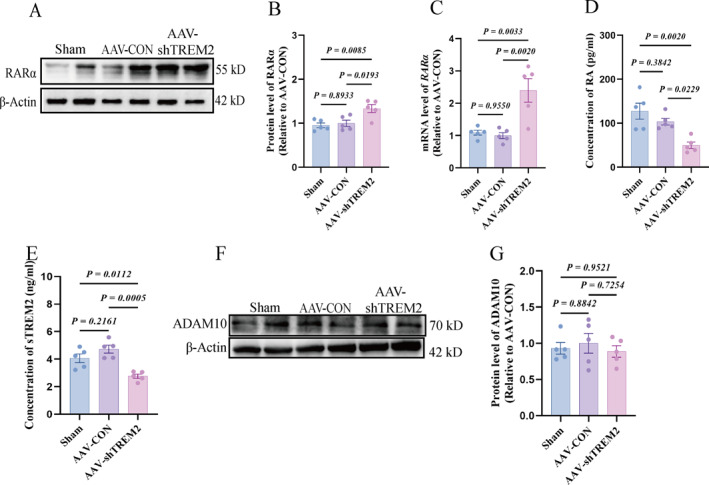
*TREM2* kd induces dysregulated expression of cerebral RA/RARα and peripheral serum sTREM2, whereas ADAM10 expression remains unaltered in the PFC. (A–B) Representative protein blot plots and quantitative analysis of RARα in PFC of three groups of rat brain, internal reference is *β*‐Actin (*n* = 5/group 95% CI “−0.3804 to 0.2696”, “−0.7705 to −0.1204”, “−0.7151 to −0.06502”). (C) The mRNA levels of *RARα* in PFC, internal reference is *GAPDH* (*n* = 5/group95% CI “−0.2391 to 0.2520”, “−0.5992 to −0.1031”, “−0.6080 to −0.1073”). (D) RA expression levels in the three group PFC (*n* = 5/group 95% CI “−22.41 to 70.01”, “31.41 to 123.8”, “7.604 to 100.0”). (E) Serum sTREM2 expression level (*n* = 5/group 95% CI “−1.654 to 0.3282”, “0.3113 to 2.294”, “0.9743 to 2.957”). (F–G) Representative protein blot plots and quantitative analysis of ADAM10 in PFC of three group rat brains, internal reference is *β*‐Actin (*n* = 5/group 95% CI “−0.4552 to 0.3176”, “−0.3431 to 0.4297”, “−0.2743 to 0.4985”). All values are shown as mean ± SEM (B–E, G); used one‐way analysis of ANOVA was used (B–E, G). AAV, adeno‐associated virus; AAV‐CON, control empty virus; AAV‐shTREM2, AAV particles containing *TREM2*; kd, knockdown; PFC, prefrontal cortex; Sham, no injection.

VPA is a well‐established teratogen that induces autism‐like behaviors in offspring via the epigenetic modulation of neurodevelopmental pathways, including RA signaling [24, 25]. Prenatal VPA exposure suppressed offspring RA/RARα signaling and downregulated *TREM2*, whereas RA supplementation restored *TREM2* expression. Chromatin immunoprecipitation confirmed RARα binding to the *TREM2* promoter, thereby supporting RA pathway regulation of TREM2. We hypothesized reciprocal TREM2 regulation of RA signaling via feedback mechanisms. The PFC RA levels decreased (Figure 5D) but *RARα* expression increased (Figure 5A–C) in *TREM2*‐kd rats, suggesting that *TREM2* negatively regulated RA‐RARα activity. Given the established cleavage of the TREM2 ectodomain into soluble TREM2 (sTREM2), which is a serum‐detectable biomarker, we quantified peripheral sTREM2 levels. The serum sTREM2 level was reduced in kd rats (Figure 5E) despite unaltered ADAM10 protease activity (Figure 5F,G), indicating that peripheral sTREM2 depletion reflected cerebral TREM2 loss and might serve as a PFC biomarker. No significant changes in peripheral inflammatory cytokines were observed (Figure S1G–J), confirming that *TREM2* downregulation induced neuroinflammation without systemic effects.

### 3.5 RA Rescued Autism‐Related Behavioral Deficits and Upregulated PFC *RARα*/*TREM2* Expression With Concomitant Elevation of Serum Level of sTREM2 in *TREM2*‐Kd Rats ‐ Mechanistic Conservation Validated In Vitro

**FIGURE 6:**
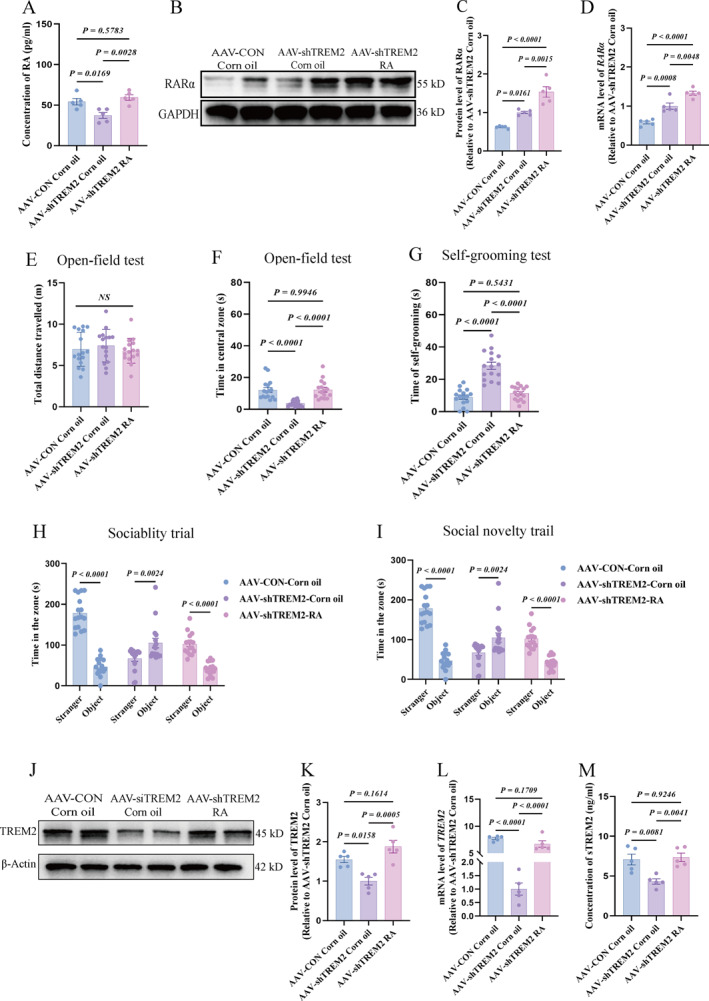
RA supplementation upregulates RA/*RARα* and *TREM2* expression in the rat PFC, elevates sTREM2 levels in peripheral blood serum, and ameliorates autism‐like behaviors in rats. (A) Three expression level of RA in brain PFC of AAV‐CON Corn oil, AAV‐shTREM2 Corn oil and AAV‐shTREM2 RA (*n* = 5/group 95% CI “3.124 to 30.55”, “−18.96 to 8.465”, “−35.80 to −8.372”). (B–C) Representative Western blotting and quantitative analysis of RARα in PFC of rat brains in three groups, the internal control is *β*‐Actin (*n* = 5/group 95% CI “−0.6846 to −0.07289”, “−1.220 to −0.6083”, “−0.8412 to −0.2295”). (D) mRNA level of *RAR* *α* in PFC, internal reference: *GAPDH* (*n* = 5/group 95% CI “−0.6437 to −0.1977”, “−0.9762 to −0.5302”, “−0.5555 to −0.1095”). (E) The total distance traveled in the open‐field test (*n* = 16/group 95% CI “−2.026 to 1.164”, “−1.412 to 1.778”, “−0.9814 to 2.209”). (F) The time spent in the central zone in the open‐field test (*n* = 16/group 95% CI “4.129 to 12.70”, “−4.458 to 4.108”, “−12.87 to −4.304”). (G) The time spent for self‐grooming in the open‐field test (*n* = 16/group 95% CI “−25.01 to −13.91”, “−7.974 to 3.119”, “11.49 to 22.58”). (H) The social interaction in the three‐chamber test (a stranger rat *vs.* an object) (*n* = 16/group 95% CI “105.0 to 158.4”, “−64.74 to −11.34”, “33.72 to 87.13”). (I) The recognition of social novelty in the three‐chamber test (a stranger rat *vs.* a familiar rat) (*n* = 16/group 95% CI “−123.9 to −80.19”, “48.99 to 95.40”, “−110.5 to −73.26”). (J–K) Representative western blot and quantification analysis of TREM2 protein in the PFC from the AAV‐CON Corn oil、AAV‐shTREM2 Corn oil and AAV‐shTREM2 RA, as normalized to *β*‐Actin (*n* = 5/group 95% CI “0.1069 to 0.9922”, “−0.7700 to 0.1153”, “−1.320 to −0.4342”). (L) The mRNA level of *TREM2* in the PFC from the three groups, as detected using RT‐qPCR and normalized to *GAPDH* (*n* = 5/group 95% CI “0.5779 to 1.167”, “0.03989 to 0.6287”, “−0.8324 to −0.2436”). (M) The protein level of sTREM2 in the serum from the three groups (*n* = 5/group 95% CI “0.7626 to 4.751”, “−2.277 to 1.711”, “−5.034 to −1.045”). The values were displayed as the mean ± SEM (A, D–I, K–L). Ordinary one‐way ANOVA was applied (A, D–I, K–M). AAV‐CON Corn oil, corn oil supplementation after empty virus injection; AAV‐shTREM2 Corn oil, corn oil supplementation after injecting *TREM2* knockdown virus; AAV‐shTREM2 RA, RA supplementation after injecting *TREM* *2* knockdown virus; PFC, prefrontal cortex; RA, retinoic acid; RT‐qPCR, enzyme‐linked immunosorbent assay.

To test the hypothesis that the RA/RARα signaling pathway regulates TREM2 and is involved in autism pathogenesis, rats with *TREM2* kd were supplemented with RA. RA supplementation significantly increased RA levels in the PFC (Figure 6A), *TREM2* expression (Figure 6J–L), and RARα levels (Figure 6B–D). Our in vitro studies demonstrated that RA supplementation upregulated *RARα* expression in si*TREM2*‐treated BV2 microglia (Figure S2M,N). Meanwhile, the expression level of serum sTREM2 was also increased (Figure 6M). The behavioral tests revealed improvements in autism‐like symptoms. shTREM2 rats with RA supplementation spent significantly more time in the central area (Figure 6F), exhibited reduced self‐grooming time (Figure 6G), and displayed significantly enhanced socialization and social novelty abilities compared with unsupplemented sh*TREM2* rats (Figure 6H,I). This indicated that RA modulated TREM2‐dependent mechanisms in autism pathogenesis and ameliorated autism‐like behaviors associated with *TREM2* deficiency.

### 3.6 RA Supplementation Attenuated Aberrant Microglial Activation and Pathological Polarization in sh*TREM2* Rats

**FIGURE 7:**
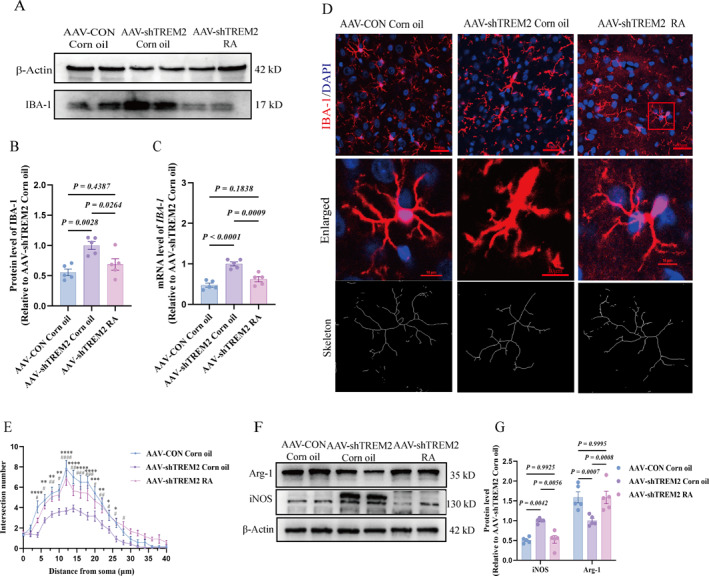
RA supplementation attenuates pathological microglial activation and restores homeostatic polarization. (A–C) IBA‐1 protein and mRNA expression levels of *IBA‐1* in PFC of three groups (*n* = 5/group 95% CI of protein “−0.7188 to −0.1680”, “−0.4063 to 0.1445”, “0.03713 to 0.5879”; 95% CI of RNA: “−0.7298 to −0.3203”, “−0.3498 to 0.05969”, “0.1752 to 0.5847”). (D) Representative immunofluorescence staining and skeleton images of IBA‐1 in the three groups of PFC (*n* = 12 slices from 4 rats per group), Scale bars = 50 and 10 μm. (E) The degree of microglia complexity. (F–G) Representative plots and statistical analysis plots of iNOS and Arg‐1 protein blots in the PFC, internal reference is *β*‐Actin (*n* = 5/group 95% CI of iNOS: “−0.8673 to −0.3256”, “−0.2903 to 0.2513”, “0.3061 to 0.8478”; 95% CI of ARG‐1: “0.1000 to 0.8735”, “−0.3833 to 0.3901”, “−0.8701 to −0.09664”). All values are shown as mean ± SEM (B–C, G); one‐way analysis of ANOVA was used (B–C, G). AAV‐CON Corn oil, corn oil supplementation after empty virus injection; AAV‐shTREM2 Corn oil, corn oil supplementation after injecting *TREM2* knockdown virus; AAV‐shTREM2 RA, RA supplementation after injecting *TREM2* knockdown virus; NS, not significant; PFC, prefrontal cortex; RA, retinoic acid. #/*p < 0.05; ##/**p < 0.01; ###/***p < 0.001; ####/****p < 0.0001.

We analyzed activation and polarization markers to evaluate the effects of RA supplementation on microglia. RA supplementation significantly reduced IBA‐1 protein and *IBA‐1* mRNA levels, indicating decreased microglial activation (Figure 7A–C). Additionally, IBA‐1 fluorescence intensity also decreased (Figure S1D–F). The morphological assessments revealed an increase in microglial branching, with longer and more numerous branches than those in the shTREM2 group without RA supplementation (Figure S3L–O). Sholl analysis confirmed a higher number of intersections, reflecting a more complex branching structure (Figure 7D,E) and enhanced microglial complexity. We next assessed microglial polarization following RA treatment. RA supplementation decreased iNOS expression while increasing Arg‐1 levels (Figure 7F,G). It also reduced *CD68* and *CD86* expression, elevated *CD206* expression (Figure S3P–R), decreased pro‐inflammatory cytokine secretion, and increased anti‐inflammatory cytokine secretion (Figure S3S–V). Collectively, these findings demonstrated that RA ameliorated microglial activation and modulated polarization.

### 3.7 RA Supplementation Normalized Synaptic Protein Expression and Synaptic Pruning

**FIGURE 8:**
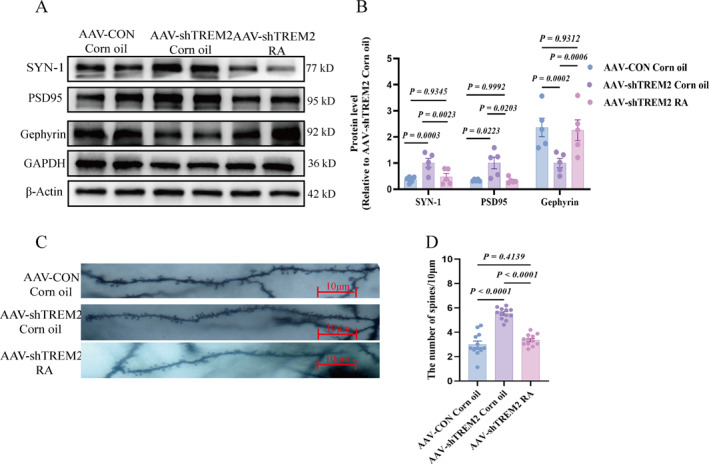
Synaptic changes in the rat PFC after RA supplementation. (A–B) Representative protein imprinting and quantitative analysis of synaptic proteins in the PFC of three groups, internal parameters GAPDH and *β*‐Actin (*n* = 5/group 95% CI of SYN‐1: “−1.565 to −0.2161”, “−0.8243 to 0.5245”, “0.06618 to 1.415”; 95% CI of Gephyrin: “0.1522 to 0.7912”, “−0.2319 to 0.4071”, “−0.7037 to −0.06460”; 95% CI of PSD95: “−0.1374 to −0.009761”, “−0.05468 to 0.07291”, “0.01888 to 0.1465”). (C) Representative dendritic spines (*n* = 12 slices from 4 rats per group), scale bar = 10 μm. (D) Dendritic spine analysis diagram. All values expressed as mean ± SEM (B, D); and applied one‐way analysis of variance (B, D). AAV‐CON Corn oil, corn oil supplementation after empty virus injection; AAV‐shTREM2 Corn oil, corn oil supplementation after injecting *TREM2* knockdown virus; AAV‐shTREM2 RA, RA supplementation after injecting *TREM2* knockdown virus; PFC, prefrontal cortex; RA, retinoic acid.

We analyzed key markers to determine whether RA supplementation normalized synaptic protein expression and pruning. RA treatment decreased SYN‐1 and PSD95 levels while increasing gephyrin expression (Figure 8A,B) and reducing dendritic spine density (Figure 8C,D), indicating restored synaptic homeostasis. Immunofluorescence revealed enhanced colocalization of IBA‐1 with PSD95 and C1q with PSD95 (Figure S2I–2L), consistent with regulated microglial pruning. Collectively, these results demonstrated that *TREM2* deficiency disrupted synaptic protein expression and pruning, whereas RA supplementation rectified these defects. This implicated the involvement of the RA/RARα signaling pathway in TREM2‐mediated ASD pathogenesis.

## 4 Discussion

ASD is a complex neurodevelopmental condition with a multifactorial etiology. Accumulating evidence highlights the role of immune activation in microglia as a key component in ASD pathogenesis. Recent studies have demonstrated that the upregulation of *TREM2*, a transmembrane receptor predominantly expressed on microglia, can mitigate aberrant microglial activation and polarization. This finding suggests a potential role of TREM2 in the etiopathogenesis of ASD. The hypothesis was investigated by administering AAV injections to normal SD rats to knock down *TREM2* expression in the brains. Following the kd, the rats exhibited hallmark of autism‐like behaviors. The behavioral deficits were accompanied by elevated microglial activation, as evidenced by significantly increased *IBA‐1* expression in the PFC. These changes were further characterized by abnormal microglial polarization, dysregulated microglia‐mediated synaptic pruning, and imbalances in excitatory/inhibitory synaptic protein expression. Furthermore, *TREM2* kd significantly reduced RA levels, with a concomitant increase in *RARα* expression in the PFC and a decrease in serum sTREM2 expression. We administered RA supplementation via gavage to rats with *TREM2* kd to assess the regulatory interplay between the RA/RARα signaling pathway and *TREM2*. RA supplementation reversed many of the pathological alterations associated with TREM2 reduction and improved autism‐like behaviors in the affected rats. Moreover, we conducted in vitro cellular experiments to investigate the effects of the RA/RARα signaling pathway on TREM2. The results demonstrated that *TREM2* kd elevated RARα levels in BV2 cells, whereas RA supplementation induced upregulation of both *TREM2* and *RARα*. These data indicated that *TREM2* mediated microglial regulation through the RA/RARα signaling pathway. In subsequent experiments, we plan to thoroughly examine the changes in the expression of RA synthesis/metabolic enzymes and analyze further mechanisms such as RARα phosphorylation or subcellular localization.

TREM2, a transmembrane protein predominantly expressed in microglia, regulates their survival, activation, phagocytosis, and migration [26]. *TREM2* deficiency impairs neuronal mitochondrial function, induces metabolic dysregulation, delays neurodevelopment, and disrupts synaptic morphology and neurotransmission [27]. Multiple studies have confirmed that TREM2 interacts with synaptic proteins to coordinate synaptogenesis and pruning, thereby modulating neuronal connectivity and signaling [28, 29, 30]. Early evidence implicating the role of TREM2 in neuropathology was provided from a study on Nasu–Hakola disease (NHD) [31], a rare disorder characterized by progressive, early‐onset dementia. Subsequent studies established the role of TREM2 in neurodegenerative diseases. However, they predominantly focused on Alzheimer's disease (AD), with few studies examining TREM2 in ASD. The postmortem analyses revealed reduced TREM2 levels in the brains of patients with ASD versus age‐matched controls [13], which was consistent with our prior findings of diminished TREM2 levels in the PFC of rats with VPA‐induced ASD [14]. *TREM2* overexpression ameliorates ASD‐like phenotypes in this model, suggesting its involvement in ASD pathogenesis. However, the underlying mechanisms need further investigation.

The traditional M1/M2 dichotomy for microglial polarization oversimplifies their dynamic, spatiotemporally specific continuum of activation states [32, 33]. Given this paradigm shift, we focused on specific molecular markers rather than categorical labels. iNOS produces nitric oxide (NO) to drive inflammation, with its upregulation correlated with disease severity in multiple pathologies [34, 35]. CD68, which is a lysosomal transmembrane glycoprotein, reflects enhanced phagocytic activity and inflammatory status [36, 37]. In our study, *TREM2* kd upregulated both *iNOS* and *CD68* in microglia, indicating a co‐occurrence of pro‐inflammatory and phagocytic activation. Substantial evidence links microglial dysregulation (e.g., elevated levels of IBA‐1 and pro‐inflammatory cytokines) to ASD models [38]. TREM2, is an innate immune receptor crucial for microglial maturation and function [5], which modulates their activation state [21]. *TREM2*‐deficient mice exhibited core ASD‐like behaviors, including social deficits and excessive self‐grooming [13], which paralleled our findings in *TREM2*‐kd SD rats. *TREM2* overexpression have been shown to rectify aberrant microglial activation in VPA‐induced ASD models [14], whereas our data demonstrated that *TREM2* kd induced pathological microglial activation (*iNOS*↑/*CD68*↑). Collectively, these results strongly suggest that *TREM2* deficiency drives ASD‐like phenotypes through dysregulated microglial activation.

Synapses are key structures facilitating information transmission between neurons in the brain [39]. Brain function is optimal when synapses have a normal structure and function. Synaptic pruning is a process pivotal in normal brain development and functional maturation, which leads to the refinement and removal of excess synapses to establish precise neuronal connections [40]. The number of synapses in the brain is higher during early childhood than during adulthood. However, a subset of these synapses form more precise synaptic connections only after they are pruned [41]. Accurate synaptic pruning is central to normal brain development because it strengthens remaining synapses [42]. However, this process is complex, and microglia can participate in synaptic pruning in the normal brain through their phagocytic activity [43]. This process is mediated by the interaction of microglial cell surface receptors with downstream signaling pathways, including TREM2 (a critical receptor in this mechanism) [13]. Our study demonstrated that TREM2 regulated the role of microglia in synaptic pruning in the developing brain. The postmortem analysis of patients with ASD revealed increased dendritic spine density, which was indicative of impaired synaptic pruning [44]. Based on these findings, we hypothesized that TREM2 may influence synaptic pruning by regulating microglial activity. The experimental results from rats with *TREM2* kd supported this hypothesis, revealing increased dendritic spine density and microglial activation with abnormal polarization. These observations strongly suggest that TREM2 is integral to maintaining proper synaptic pruning via microglial regulation.

Besides microglia, other synapse‐associated proteins, such as SYN‐1, PSD95, and gephyrin, play key roles in maintaining synaptic structure and function [45]. SYN‐1 is involved in synaptic vesicle membrane processes and neurotransmitter release [46], whereas PSD95, an excitatory postsynaptic protein, interacts with various ion channels, receptors, and signaling molecules [47]. It facilitates synaptic stability and signal transduction. On the contrary, gephyrin, which is an inhibitory postsynaptic protein, anchors glycine and GABA receptors, thereby maintaining inhibitory synaptic balance [48]. Studies on VPA‐exposed rats [14] have demonstrated dysregulation of presynaptic and postsynaptic structural and functional proteins, suggesting their importance in maintaining synaptic integrity. TREM2 has been shown to influence synaptic plasticity by regulating synaptic protein expression [29]. For example, *TREM2* overexpression ameliorated synaptic protein dysregulation in models of anesthesia‐ and surgery‐induced cognitive decline in aging mice [49]. In our study, *TREM2* kd in normal rats led to the dysregulation of these critical presynaptic and postsynaptic proteins and altered dendritic spine density, thus reinforcing the connection between TREM2 activity and synaptic regulation. Both the classical complement cascade and TREM2 have been implicated in microglia‐mediated synaptic pruning [50]. The complement system comprises three activation pathways: classical, alternative, and lectin [51]. Complement proteins C1q and C3 have been shown to mediate synaptic clearance by microglia [52], with C3 playing a central role in activating all three pathways [53]. CR3A, which binds to C3 with high affinity, is highly expressed in microglia and facilitates their synaptic pruning function [54]. Our study found that *TREM2* kd significantly reduced C3 and CR3A expression, but with no significant abnormality in the number of neurons, suggesting that the TREM2‐C3/CR3A axis is a key pathway for microglia‐mediated synaptic pruning.

Furthermore, the loss‐of‐function (LoF) mutations in *TREM2* play an important role in neurodegenerative diseases, particularly AD. Impaired TREM2 function diminishes the microglial response to Aβ plaques, thus reducing peri‐plaque clustering and exacerbating neuritic dystrophy [55]. This loss of plaque surveillance accelerates Aβ deposition and neuronal dystrophy, highlighting the crucial role of TREM2, in coordinating protective microglial responses. Additionally, *TREM2* deficiency compromises the phagocytic clearance of apoptotic neurons and protein aggregates. Ulland et al. [56] demonstrated that TREM2‐mediated phagocytosis of neuronal debris was essential for CNS homeostasis; its impairment contributed to synaptic loss and heightened neuroinflammation. Moreover, TREM2 mutations exacerbated tau pathology. In tauopathy mouse models, *TREM2* deficiency impeded microglial uptake of tau fibrils, accelerating transneuronal tau spread [57]. Concurrently, TREM2 loss promoted a shift in microglia toward a pro‐inflammatory phenotype. Keren‐Shaul et al. identified [58] a TREM2‐dependent disease‐associated microglia (DAM) phenotype that conferred a neuroprotective state. The absence of TREM2 blocked DAM activation, skewing microglia toward a maladaptive inflammatory state characterized by the release of cytotoxic cytokines (e.g., IL‐1β, TNF‐α), ultimately amplifying neuronal damage. Human studies corroborated these mechanisms. The *TREM2 R47H* variant was initially linked to AD by Guerreiro et al. [59], with studies demonstrating elevated CSF tau levels and accelerated cognitive decline in carriers. Although increasing evidence connects *TREM2* LoF mutations to AD pathogenesis via microglial dysfunction, a direct association with ASD risk remains unestablished. Large‐scale genetic studies in ASD populations have not consistently identified TREM2 as a major susceptibility locus. However, given the key role of microglia in neurodevelopment and reported cases of *TREM2* variants in patients with neurodevelopmental disorders, further investigations into the potential contribution of *TREM2* dysregulation, including LoF mutations, to ASD or related phenotypes are warranted.

Emerging therapeutic approaches focus on modulating the *TREM2* pathway as a potential therapeutic target for neurological disorders. Novel interventions regulating *TREM2* expression and its downstream signaling pathways can potentially mitigate pathological conditions linked to synaptic dysfunction. RA levels are reduced in pediatric patients with ASD and inversely correlated with symptom severity [18]. RA plays a pivotal role in neurodevelopment by binding to RARα, which is predominantly expressed in the cerebral cortex and hippocampus. RARα has been identified as a key regulator of microglial activity. Smadar Goldfar et al. conducted an unbiased analysis of microglial regulatory sequences [20], detecting significant enrichment of RARα‐associated motifs. Animal and population studies have linked the downregulation of the RA/RARα signaling pathway to ASD pathogenesis, although the precise mechanisms remain unclear [60].

Our previous studies demonstrated that prenatal VPA exposure induced autism‐like behaviors in offspring by suppressing RA/RARα signaling, downregulating *TREM2* expression, and activating microglia [14, 15]. We further established that RARα directly bound to the *TREM2* gene promoter and positively regulated its transcriptional activity, thereby identifying RARα as an upstream transcriptional regulator of *TREM2*. Building on this, we assessed key RA/RARα pathway molecules in the PFC of *TREM2*‐kd rats. The results showed that *TREM2* kd significantly reduced local RA levels but unexpectedly increased RARα protein expression. Given the observed transcriptional regulation of TREM2 by RARα, we hypothesized that *TREM2* might negatively regulate *RARα* expression through a feedback mechanism. The loss of *TREM2* expression might attenuate this negative regulation, leading to *RARα* upregulation; however, the specifics of this putative mechanism require further investigation. We administered exogenous RA to *TREM2*‐kd rats to explore the role of the RA/RARα pathway in the behavioral phenotypes of the *TREM2* deficiency model. The intervention results showed that RA supplementation effectively reversed the reduced brain RA levels and significantly ameliorated repetitive/stereotyped behaviors and social deficits in these rats. Concurrently, RA supplementation markedly suppressed microglial activation, attenuating their pro‐inflammatory phenotype and enhancing an anti‐inflammatory state, thus aligning with previous reports showing that RA inhibited microglial activation and modulated their functional polarization [61]. Integrating these findings with our previous studies, we concluded that prenatal VPA exposure potentially downregulated *TREM2* expression by inhibiting RA/RARα signaling. This *TREM2* deficiency disrupted RA/RARα homeostasis (characterized by reduced RA and compensatory RARα upregulation), subsequently driving aberrant microglial activation and functional polarization dysregulation. These alterations might ultimately contribute to abnormal synaptic pruning and collectively underlie the core autism‐like behavioral symptoms observed in the experimental rats. The RA/RARα‐TREM2 axis represents a crucial molecular node governing microglial function and autism‐like neurodevelopmental abnormalities.

The *TREM2* gene encodes a transmembrane immunoglobulin receptor comprising a signal peptide sequence, an exon, a transmembrane region, and an extracellular structural domain [62]. Its extracellular structural domain can be cleaved by the protein hydrolase ADAM10/ADAM17 to produce sTREM2, which is detectable in serum [63]. Studies indicate that ADAM17 acts as the primary sheddase for TREM2 under physiological conditions [64]. For instance, upregulated ADAM17 activity reduced *TREM2* expression and exacerbated neuroinflammation in a lead exposure‐induced hypertension mouse model [65]. Furthermore, iRhom2, a noncatalytic subunit of ADAM17, influences TREM2 shedding efficiency by modulating ADAM17 stability [64]. However, ADAM10 may become predominant in specific contexts such as neuroinflammatory stimulation [66], thus directly mediating TREM2 proteolysis. In *C9orf72*‐related ALS/FTD models, *NLRP3* inflammasome activation and subsequent IL‐1β production significantly enhanced ADAM10‐mediated TREM2 cleavage, elevating sTREM2 levels [67]; this cleavage potentially disrupted TREM2 immunomodulatory functions. Within AD, *ADAM10* genetic variants are associated with altered TREM2 shedding, where specific risk alleles reduce cell surface ADAM10 expression and modify sTREM2 release kinetics [68]. Consequently, serum sTREM2 levels may be influenced by ADAM10 abundance, with reduced ADAM10 potentially contributing to lower serum sTREM2 levels. Correlations exist between CSF and serum sTREM2 levels in AD [69], supporting the potential of sTREM2 as a biomarker [70]. In our study using *TREM2*‐kd rats, decreased brain *TREM2* expression coincided with reduced serum sTREM2 levels and the emergence of autism‐like behaviors. In contrast, RA supplementation increased brain *TREM2* expression, elevated serum sTREM2 levels and ameliorated autism‐like behaviors. ADAM10 levels remained unchanged following *TREM2* kd, indicating that the decline in peripheral serum sTREM2 was attributable to reduced CNS *TREM2* expression rather than altered shedding efficiency. Overall, these findings suggest serum sTREM2 as a potential biomarker candidate for ASD, warranting further validation. Our study aimed to provide a novel method for early ASD identification in children and explored TREM2‐related therapeutic strategies, potentially revealing new treatment avenues for ASD.

Our study systematically investigated for the association between *TREM2* and ASD, with a focus on the relationships among TREM2, microglial activation, synaptic pruning, and the RA/RARα signaling pathway, aiming to define the underlying mechanisms. Although our findings demonstrated that the RA/RARα pathway, via TREM2, contributed to microglial activation and synaptic pruning, whether *TREM2* functions exclusively within microglia remains unclear. RA/RARα signaling has been shown to modulate not only microglia but also astrocyte and neuronal function [71, 72], and the alterations in astrocyte and neuronal activity can subsequently influence microglial activation [73]. Therefore, a key question to be addressed in future investigation is whether the RA/RARα signaling pathway can indirectly regulate microglial activation by influencing complex cross‐cellular interactions involving neurons and glia. Furthermore, we identified a positive correlation between serum sTREM2 levels and TREM2 expression in the PFC. However, the exact source of circulating sTREM2, particularly its potential origin from the brain, remains to be defined and warrants further investigation. Previous studies [74] have demonstrated that TREM2 signaling primarily modulates localized microglial activity within the CNS, with no established evidence supporting its role in systemic inflammatory processes. However, immune dysregulation and neuroinflammation contribute to ASD pathogenesis. Systemic inflammation (e.g., chronic inflammation triggered by maternal immune activation or early‐life infections) during crucial developmental windows may impact neurodevelopment through indirect mechanisms, such as metabolic dysfunction transmitted across the blood–brain barrier. This process can induce core phenotypes resembling ASD [75, 76]. Furthermore, the role of TLR‐4 signaling in amplifying inflammatory responses highlights its potential as a therapeutic target [77]. Modulating this pathway can alleviate ASD‐associated neuroinflammation, independent of peripheral immune activity. Our study showed that reduced *TREM2* levels induced neuroinflammation, whereas peripheral inflammatory mediators showed no significant alterations. This dissociation potentially reflected the compartmentalization of neuroimmune responses within the CNS. However, further research is still needed to explore the differences between *TREM2* gene knockout heterozygous mice and mice with reduced *TREM2* expression only in the brain need further investigation. Additionally, the relationship between *TREM2* dysregulation, systemic inflammation, and neuroimmunity should be explored to further reveal ASD pathogenesis.

Our study had certain limitations. Focusing on the PFC and on microglia which exhibited no connectivity changes in other brain regions, might have obscured broader insights. Given the higher prevalence of ASD in men in clinical settings [78], and the greater documented propensity of male rats to exhibit autism‐like behaviors [79], this study was conducted exclusively in male offspring, thereby precluding insights into potential sex‐related effects. In addition, we focused on the C3 and CR3A signaling pathways, which represent one component of the complement system. Future research should investigate the roles of other complement components, such as C1q and C4 to provide a more comprehensive understanding of the molecular mechanisms underlying TREM2‐mediated synaptic pruning.

## 5 Conclusions

The present study demonstrated that *TREM2* kd in normal rats induced abnormal microglial activation, polarization, and synaptic pruning, culminating in autistic‐like behaviors. RA supplementation restored microglial function and synaptic pruning, improving these autistic‐like behaviors. These findings positioned TREM2 as a key regulator of microglial activity and synaptic pruning. The *TREM2* gene expression can be upregulated by activating RARα via RA supplementation, offering a novel therapeutic avenue for ASD treatment. The proteolytic shedding of the TREM2 ectodomain generates sTREM2, which may be correlated with cerebral TREM2 expression levels. Preliminary evidence suggests that the peripheral serum level of sTREM2 can potentially serve as a candidate biomarker for early‐stage ASD, highlighting its potential in refining diagnostic strategies and guiding therapeutic development.

## Author Contributions

M.L. conducted the experiments, analyzed the data and drafted original manuscript. K.B. and Y.B. helped to conduct behavior tests. Y.M. provided technical guidance during the study. T.Z. and Y.Y. helped collect samples and provided statistical analysis methodology. T.L. and J.C. supported experimental design and supervision. T.Y. revised the manuscript. H.W. designed whole study, revised original manuscript, as well as provided financial support. All authors approved the final manuscript.

## Ethics Statement

The animal ethics committee of the Children's Hospital of Chongqing Medical University (CHCMU‐IACUC20240111004) approved the experimental protocol.

## Consent

The authors have nothing to report.

## Conflicts of Interest

The authors declare no conflicts of interest.

## Supporting information


Supporting Information S1

**Figure S1**: Effects of *TREM2* kd combined with RA supplementation on microglial cellular density and fluorescence intensity expression and systemic inflammatory cytokine levels following *TREM2* kd.

**Figure S2**: Co‐expression patterns of IBA‐1^+^/TREM2^+^, IBA‐1^+^/PSD95^+^, and C1q^+^/PSD95^+^ in microglia following *TREM2* kd and RA supplementation.

**Figure S3**: Kd of *TREM2* triggers aberrant microglial activation and polarization, whereas RA supplementation rescues these pathological alterations.

## Data Availability

All data supporting the findings of this study were available within the article and its Supplementary Information files or from the corresponding author upon reasonable request.
