# Supplementary material for: TREM2 Downregulation Disrupts Microglial Function and Synaptic Pruning Through RA/RARα Signaling: Mechanisms Underlying Autism‐Like Behaviors
Source: Pediatr Discov. 2025 Oct 16;3(4):e70024. doi: 10.1002/pdi3.70024 (PMC12753026; doi:10.1002/pdi3.70024)
Supplement: Supplementary file 1 — Supporting Information S1 [file PDI3-3-e70024-s001.docx]

**Table 1**

**Antibodies used for western blotting in this study.**

| Target protein | Catalog | Host | Vendor | Dilution |
| --- | --- | --- | --- | --- |
| TREM2 | 510482 | Rb | Zenbio | 1:500 |
| IBA-1 | 26177-1-AP | Rb | Proteintech | 1:1000 |
| INOS | GTX130246 | Rb | Genetex | 1:1000 |
| ARG-1 | 16001-1-AP | Rb | Proteintech | 1:1000 |
| SYN-1 | 5297T | Rb | Cell Signaling Technology | 1:1000 |
| PSD95 | 3450S | Rb | Cell Signaling Technology | 1:1000 |
| Gephyrin  RARα  C3  CR3A  GAPDH  β-Actin | 14304S  GTX54703  21337-1-AP  49692  GTX100118-S  66009-1-Ig | Rb  Rb  Rb  Rb  Rb  Mo | Cell Signaling Technology  Genetex  Proteintech  Signalway Antibody  Genetex  Proteintech | 1:1000  1:1000  1:1000  1:1000  1:10000  1:10000 |

**Table 2**

**Sequences of the specific primers for rat genes used in Real-Time Quantitative Polymerase Chain Reaction.**

| Gene name | Forward primer (5′−3′) | Reverse primer (5′−3′) |
| --- | --- | --- |
| *TREM2* | ACAGCGACCAGAACCGTAGT | ACAGAGGTATCTAGCAGGTCAGGG |
| *RARα* | AGATCACGGACCTTCGGAGTATCAG | CCTGGATAAGTGGTGGCATGGAAC |
| *IBA-1* | ATGCCCTCCTTCTGTATCTTCTACCC | CCATTGCCATTCAGATCAAACTCCATG |
| *ARG-1* | AGAGGAGGTGACTCGTACTGTGAAC | TCTGGCTTATGATTACCTTCCCGTTTC |
| *CD68* | CTCTCTTGCTGCCTCTCATCATTGG | GCTGGTAGGTTGATTGTCGTCTCC |
| *CD86* | CTCATCTAAGCAAGGATACCCGAAACC | GAATGGAAGAGATAGGCTGATGGAGAC |
| *CD206*  *GAPDH* | CTCCCTCAATGGAACACACACT  CCTGGAGAAACCTGCCAAG | TCCAAGAGTTGAACAGCGACC  CACAGGAGACAACCTGGTCC |
