# Supplementary figures and images for: TREM2 Downregulation Disrupts Microglial Function and Synaptic Pruning Through RA/RARα Signaling: Mechanisms Underlying Autism‐Like Behaviors
Source: Pediatr Discov. 2025 Oct 16;3(4):e70024. doi: 10.1002/pdi3.70024 (PMC12753026; doi:10.1002/pdi3.70024)

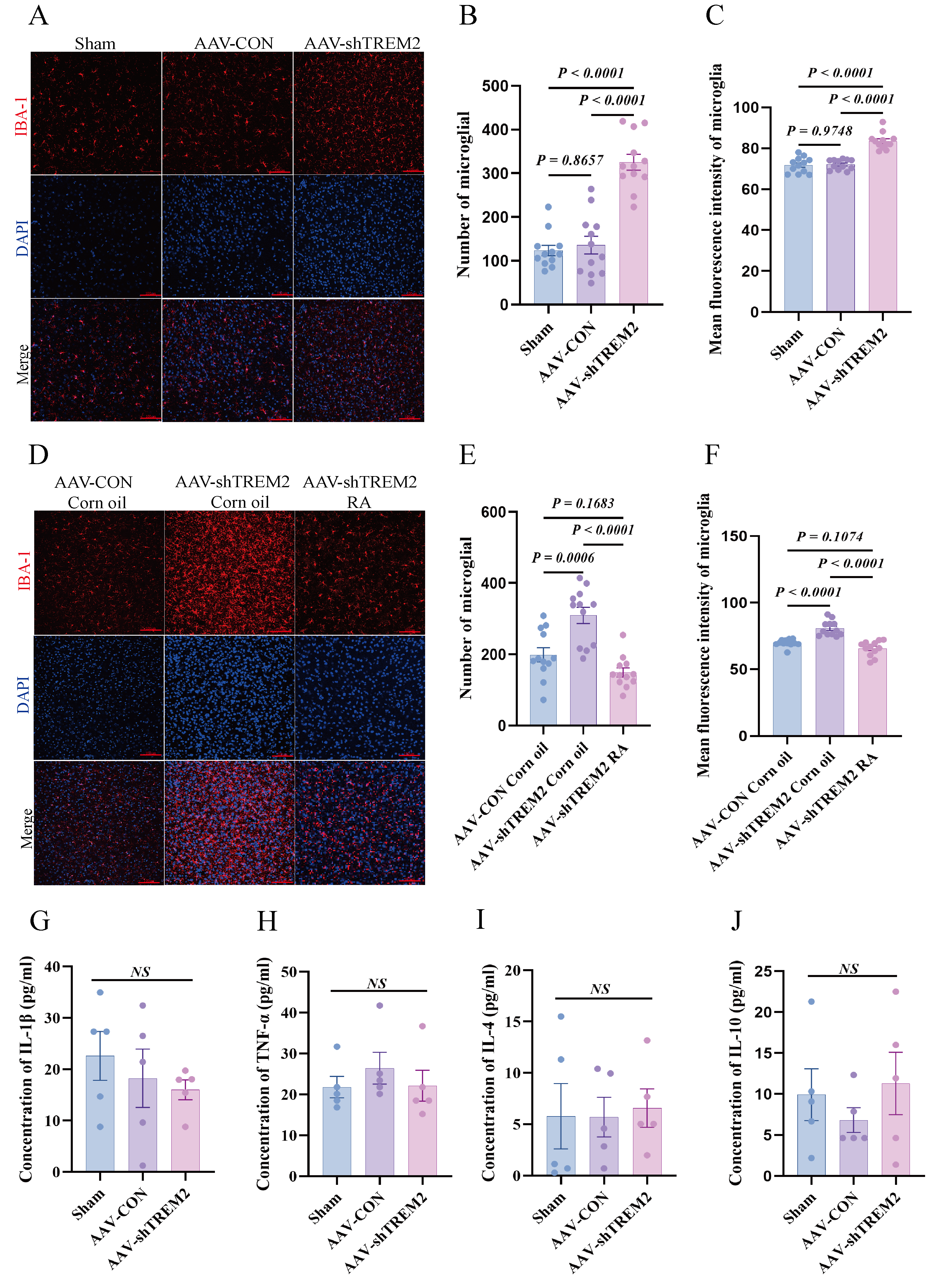

Supplement: Supplementary file 2 — Figure S1: Effects of TREM2 kd combined with RA supplementation on microglial cellular density and fluorescence intensity expression and systemic inflammatory cytokine levels following TREM2 kd. [file PDI3-3-e70024-s003.tif]

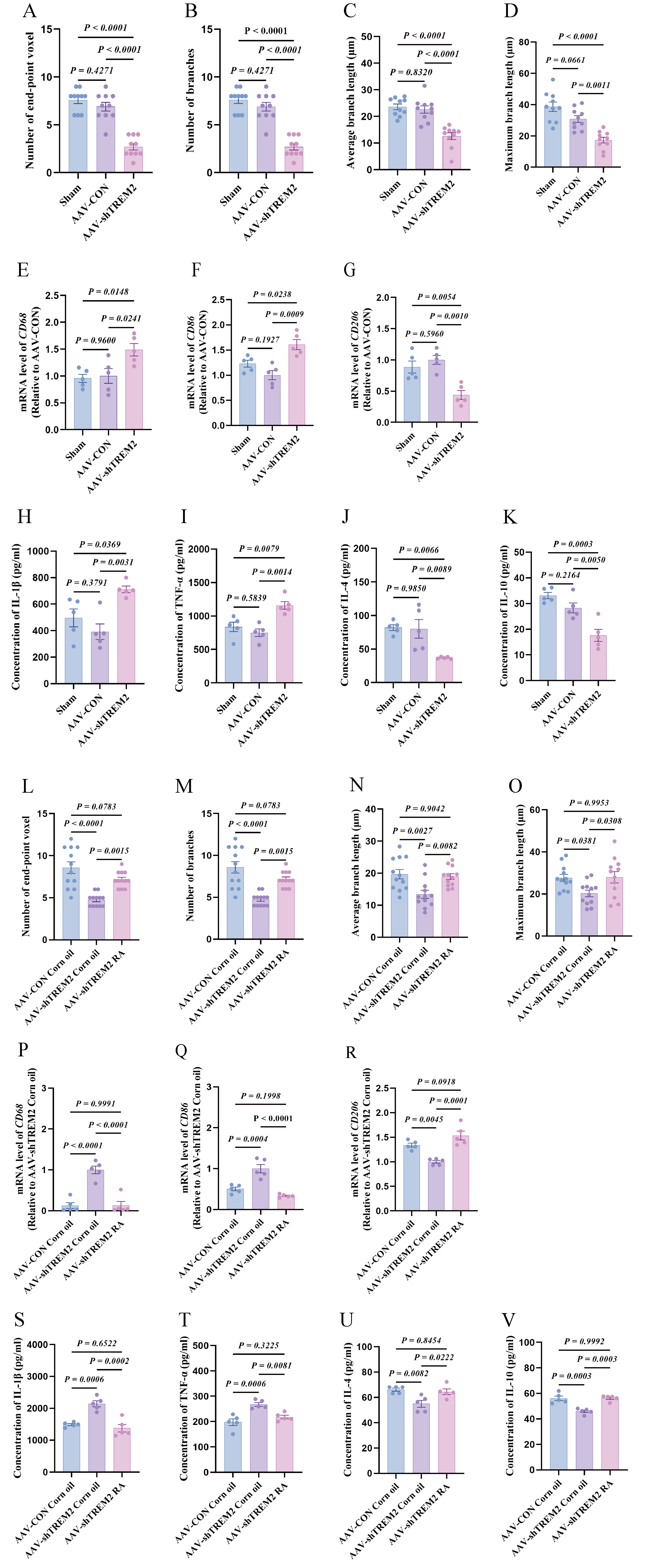

Supplement: Supplementary file 4 — Figure S3: Kd of TREM2 triggers aberrant microglial activation and polarization, whereas RA supplementation rescues these pathological alterations. [file PDI3-3-e70024-s002.tif]
